# Exploring Musical Feedback for Gait Retraining: A Novel Approach to Orthopedic Rehabilitation

**DOI:** 10.3390/healthcare13020144

**Published:** 2025-01-14

**Authors:** Luisa Cedin, Christopher Knowlton, Markus A. Wimmer

**Affiliations:** Department of Orthopedic Surgery, Rush University Medical Center, 1611 W Harrison Street, Suite 201, Chicago, IL 60612, USA; luisa_cedin@rush.edu (L.C.); christopher_knowlton@rush.edu (C.K.)

**Keywords:** biofeedback, sonification, gait retraining, plantar pressure

## Abstract

Background/Objectives: Gait retraining is widely used in orthopedic rehabilitation to address abnormal movement patterns. However, retaining walking modifications can be challenging without guidance from physical therapists. Real-time auditory biofeedback can help patients learn and maintain gait alterations. This study piloted the feasibility of the musification of feedback to medialize the center of pressure (COP). Methods: To provide musical feedback, COP and plantar pressure were captured in real time at 100 Hz from a wireless 16-sensor pressure insole. Twenty healthy subjects (29 ± 5 years old, 75.9 ± 10.5 Kg, 1.73 ± 0.07 m) were recruited to walk using this system and were further analyzed via marker-based motion capture. A lowpass filter muffled a pre-selected music playlist when the real-time center of pressure exceeded a predetermined lateral threshold. The only instruction participants received was to adjust their walking to avoid the muffling of the music. Results: All participants significantly medialized their COP (−9.38% ± 4.37, range −2.3% to −19%), guided solely by musical feedback. Participants were still able to reproduce this new walking pattern when the musical feedback was removed. Importantly, no significant changes in cadence or walking speed were observed. The results from a survey showed that subjects enjoyed using the system and suggested that they would adopt such a system for rehabilitation. Conclusions: This study highlights the potential of musical feedback for orthopedic rehabilitation. In the future, a portable system will allow patients to train at home, while clinicians could track their progress remotely through cloud-enabled telemetric health data monitoring.

## 1. Introduction

Musculoskeletal conditions on the lower extremities, such as osteoarthritis, fracture, and joint instability, often lead to gait impairments [1,2,3]. Walking is a fundamental functional activity and an important indicator of general health. Therefore, these impairments will significantly impact the patient’s mobility, overall functional capacity, and quality of life [4].

Gait training is widely used in orthopedic rehabilitation to address specific biomechanical concerns and improve the walking pattern. The primary goal varies according to the musculoskeletal disorder and the individualized aspects of the patient. For example, in patients with knee osteoarthritis, the end goal is to reduce joint loading and alleviate pain [2]. For lower limb arthroplasties, one concern is symmetry between sides, reflecting step-length and stance-time discrepancies [5,6]. For patients with chronic ankle instability, the target is the altered center of pressure (COP) location, which increases their risk of ankle sprain [7]. Gait training can also be used as a preventive tool for healthy individuals to avoid overuse running injuries, such as patellofemoral pain syndrome and tibial stress injuries [8,9].

Auditory, visual, and haptic feedback are emerging strategies that support patients to independently perceive and adjust their performance [1,2,5,8,10]. While visual feedback is commonly utilized in research [8], auditory feedback can be delivered continuously without constraining body movement or visual focus, making it a more suitable option for field applications. The translation of movement data into sound, known as movement sonification, is clinically relevant because it facilitates rapid motor learning and allows daily home-based training [10,11,12]. The auditory system’s ability to properly perceive rhythm, meter, and time-dependent changes enhances the effectiveness of auditory feedback, particularly in gait-related interventions [13,14,15]. Additionally, a study showed that auditory feedback improved the integration of the proprioceptive sensory system, with gradual increases in its reliance, further contributing to motor learning [16].

Research on auditory feedback has increased over the past years, but only a few applied it to orthopedic conditions. Auditory feedback has been well accepted by patients with joint replacement, and it significantly changed patients’ gait parameters—speed, stride length, and stance phase [5,14,17]. Our team recently published a case study that employed auditory feedback to enhance ankle fracture post-surgery rehabilitation, showing improvements in foot rollover and total force distribution [18]. Previously, two studies from our lab successfully used auditory feedback to medialize plantar pressure through an auditory cue and indirectly reduce medial knee joint loading in healthy individuals [19,20]. Preliminary data from a clinical trial (NCT02955225) show that the same system is able to medialize plantar pressure and reduce medial knee loading in patients with knee osteoarthritis, but to a much lesser extent [21]. A similar auditory system was successfully applied to patients with chronic ankle instability [22]. In this context, further research is necessary to comprehend the potential of auditory feedback on orthopedic rehabilitation.

Personalization of biofeedback plays an important role in the treatment’s success. Real-time biofeedback is often applied, as it is tailored to the patient’s condition, addressing necessary changes in performance or in the results [10]. Music also contributes to the personalization of rehabilitation while improving motor learning. Furthermore, music is known to encourage exercise and long-term rehabilitation by activating reward- and emotion-associated neural systems and eliciting pleasurable sensations [23,24,25]. Music has been used to gait retrain runners [26], but it has not yet been employed with orthopedic clinical populations.

Differing from previous studies, we employed sophisticated sonification that applies biofeedback in the form of alterations to music, shifting to a more appealing and engaging mode of feedback to a future clinical population. In addition to describing the technical approach, our main goal in this study was to understand whether healthy individuals shift their center of pressure (COP) medially while walking based solely on musical feedback without specific verbal instructions. Further, we evaluated changes to walking spatiotemporal parameters, as well as participants’ qualitative ratings of the musical feedback system. We hypothesized that the participants would shift their gait line medially when cued by musical feedback.

## 2. Materials and Methods

### 2.1. Study Design

**Figure 1:**
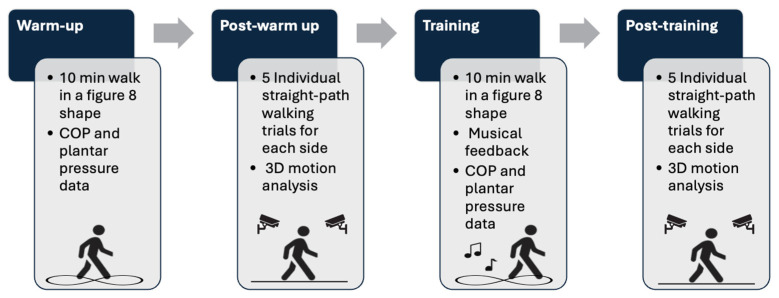
Flow chart of the study design with descriptions of data collected in each condition. COP: center of pressure.

We performed a pilot study to test the efficacy of the proposed musical feedback system, comparing gait data at four time points: warm-up, the post-warm-up baseline, during training, and post-training. Once consented, the participants were asked to choose songs from a playlist of popular songs (see Supplementary Material S1) based on their preferences and those they found most motivating for exercise. Then, the participants were fitted for the correct size of a wireless pressure-sensing insole (Insole3–Moticon, OpenGo, Munich, Germany) and a standardized test shoe (Adidas VRX Low model DB3176, Adidas, Portland, OR, USA). The participants walked overground in an elongated figure-8 shape approximately 12 m long by 3 m wide for ten minutes to warm up the insoles and collect baseline COP data. After warming up, individual straight-path walking trials without musical feedback were performed to track participants’ movements using a 23-camera optical motion capture system (Qualisys AB, Gothenburg, Sweden) and pressure-sensing insoles. Before the training session, the participants were instructed on the musical feedback to avoid muffling the sound, and they were encouraged to walk in a way that would be perceived as normal from another person’s perspective. When participants were confident that they understood the musical feedback, they were asked to practice walking with the feedback along the elongated figure-8 path for ten minutes. After training with the musical feedback, participants repeated the individual straight-path walking trials with the optical motion capture system without musical feedback. See details of the study design in Figure 1.

### 2.2. Participants

Twenty healthy subjects (9 females, 11 males, 29 ± 5 years old, 75.9 ± 10.5 kg, 1.73 ± 0.07 m) were recruited inside and outside the Rush community and tested at the Motion Analysis Laboratory at RUSH University Medical Center. Participants were eligible if they were aged 18 to 75 years old, had no history of hip, knee, or ankle surgery, had no lower extremity joint pain during ambulation, had no diagnosis of musculoskeletal injury or disease in the past 3 months, were able to walk 5 m (14 foot) intervals on leveled surfaces without significant fatigue, and were able to walk without an assistive device such as a walker or cane. Due to the musical component of the study, we only included participants with no hearing impairments. The study was approved by the University’s Institutional Review Board (ORA: 22081709-IRB01), and all procedures followed the principles of the Declaration of Helsinki [27].

Power analysis was performed using G*Power (version 3.1.9.6 Kiel University, Kiel, Germany) [28] to determine the required sample size. Based on the He et al. 2019 [20] study with healthy subjects and auditory feedback, an effect size of f = 1.16 was determined. To achieve 95% power (β = 0.95), with a shared sample across pre- and post-training groups, the analysis indicated that 10 participants would be required. In this study, we enrolled 20 participants to account for the unknown effect of music and any dropouts that may be encountered.

### 2.3. Gait Line and Plantar Pressure

**Figure 2:**
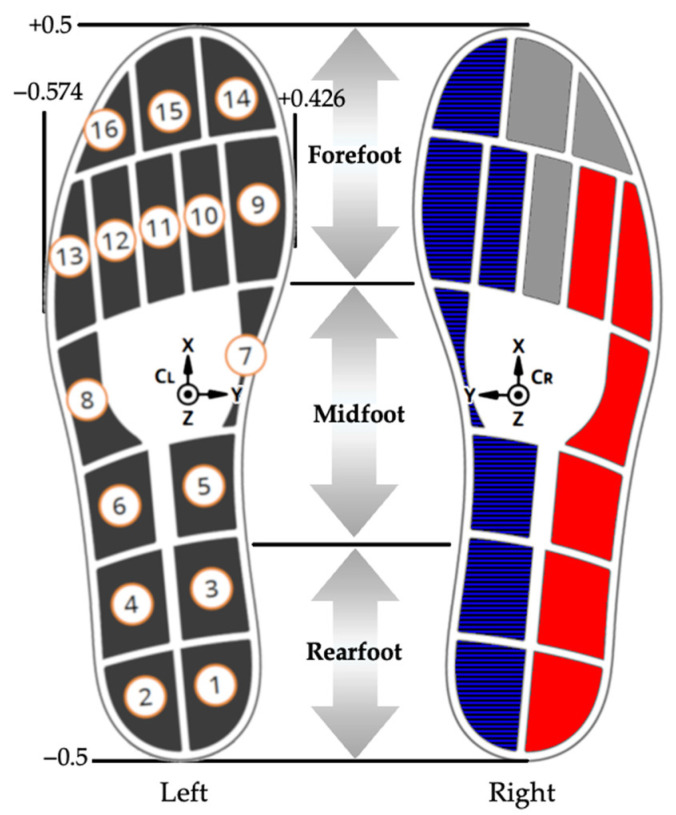
Representation of insoles’ geometry, sensors’ locations, medial (blue-colored sensors) and lateral (red-colored sensors) boundaries, and IMU positioning. Adapted from the manufacturer’s user guide (Insole3–Moticon, OpenGo, Munich, Germany).

Plantar pressure and COP were captured from a pair of wireless pressure-sensing insoles (Insole3–Moticon, OpenGo, Munich, Germany) containing 16 sensors and a six-axis inertial measurement unit (IMU). Data were obtained at 100 Hz during warm-up and training for five minutes in each condition after calibration, according to the manufacturer’s instructions. For lateral plantar pressure characterization, we summed the values obtained from sensors 2, 4, 6, 8, 12, and 13. Plantar pressure data from sensors 1, 3, 5, 7, 9, 10, and 14 were combined for medial plantar pressure determination (see Figure 2 for the sensors’ position).

Anterior–posterior COP (COPx) values were recorded as a fraction of the total length of each insole; medial–lateral COP (COPy) values were recorded as a fraction of the total width of the insole. Per the manufacturer, the origin of the COP values and the location of the 16 capacitive sensors are shown in Figure 2.

The foot was also divided into the forefoot, midfoot, and rearfoot to determine the position in which the average peak COP occurred at the baseline and after training. The first 35% of the insole length (anterior–posterior distance) represented the forefoot, the following 35% comprised the midfoot, and the remaining portion (30%) was the rearfoot.

### 2.4. Musical Feedback

**Figure 3:**
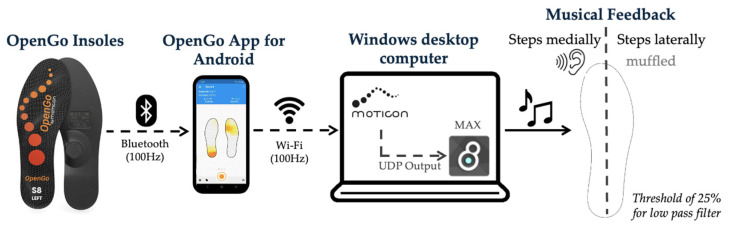
Musical feedback design.

The music was played from an individually selected playlist of popular songs over speakers in the lab. To provide real-time feedback for the intended shift in COP, plantar pressure data were sent from the insoles to a smartphone app (OpenGo, version 03.12.01) via Bluetooth connection and then transmitted via Wi-Fi to a desktop application (Moticon OpenGo Software, version 03.08.01). From this program, data were sent via the User Datagram Protocol (UDP) to Max 8 (Cycling ’74, Walnut, CA, USA), a visual, object-based programming environment for music and multimedia, which applied a lowpass filter to the music when the mediolateral COP trajectory exceeded 75% of the subject’s baseline (see Figure 3 for more details). Applying a lowpass filter with a cut-off frequency of 500 Hz in real time to music was heard by the participants as a muffling of the sound. As the subject further exceeded the COP threshold, the threshold of 75% for the filter continued to lower, muffling the sound more. Both insoles were sonified; therefore, the participant received real-time musical feedback for both sides while walking. The subjects were instructed to avoid muffling the sound while maintaining a comfortable, natural-feeling gait. These minimal instructions allowed the subjects to self-select any combination of gait alterations to achieve a medialized pressure pattern. The goal of the musical feedback was to medialize the plantar pressure, which would medialize the COP. See Supplementary Video S1 for more details on the musical feedback.

### 2.5. Spatiotemporal Parameters and Gait Cycle

A 23-camera, three-dimensional (3D) motion capture system (Qualisys AB, Gothenburg, Sweden) recording at 120 Hz was used to measure the spatiotemporal parameters and identify the gait cycle. Motion data were collected during single walking trials after warming up and after training with the musical feedback. The participants were asked to walk at a self-selected speed on a 6 m level walkway to complete five trials for each side. A modified Helen Hayes marker set [29,30] was adopted, in which 31 retroreflective markers were affixed to both sides at the posterior calcaneus, lateral calcaneus, fifth metatarsal head, second metatarsal head, lateral malleolus, medial malleolus, lateral and medial joint lines, anterior aspect of mid-thigh, greater trochanter, anterior and posterior iliac spines, and iliac crest. Markers were also placed at the sacrum (L4–L5), manubrium, and vertebra prominens at the neck (C7). The motion capture system integrated two inground force plates (Bertec Corporation, Columbus, OH, USA) that participants walked over during individual straight-path walking trials and were used in heel-strike detection (and thus gait event determination). Gait data were labeled through Qualisys AB and processed using Visual3D (HAS-Motion, Kingston, ON, Canada). Both sides were analyzed before and after feedback implementation.

### 2.6. Qualitative Rating

After the completion of the session, the participants were asked to respond to an ad hoc user experience questionnaire about their experience with the provided musical feedback. The questionnaire covered aspects related to comprehension, perceptibility, enjoyment, physical demand, discomfort, and potential to adopt as a treatment tool. It consisted of ten questions based on a Likert scale (strongly disagree, disagree, neither agree nor disagree, agree, and strongly agree) and two open-ended questions. See the complete questionnaire in Supplementary Material S2.

### 2.7. Statistical Analysis

Statistical analysis was performed using MATLAB (R2023a, Mathworks, Natick, MA, USA). A paired Student’s *t*-test was used to compare plantar pressure data before and after the implementation of musical feedback. The comparison was performed between warm-up and training (walking for 10 min in a figure-8 shape), as well as post-warm-up and post-training (single walking trials). Differences in gait lines for both sides were identified using one-dimensional statistic parametric mapping (SPM). The significance level was set to 0.05. Warm-up and baseline medial–lateral changes throughout the gait line were compared as a percentage of difference (delta), and the peak change was identified and cross-checked with the region of the foot where it occurred.

## 3. Results

### 3.1. Gait Line and Plantar Pressure

**Figure 4:**
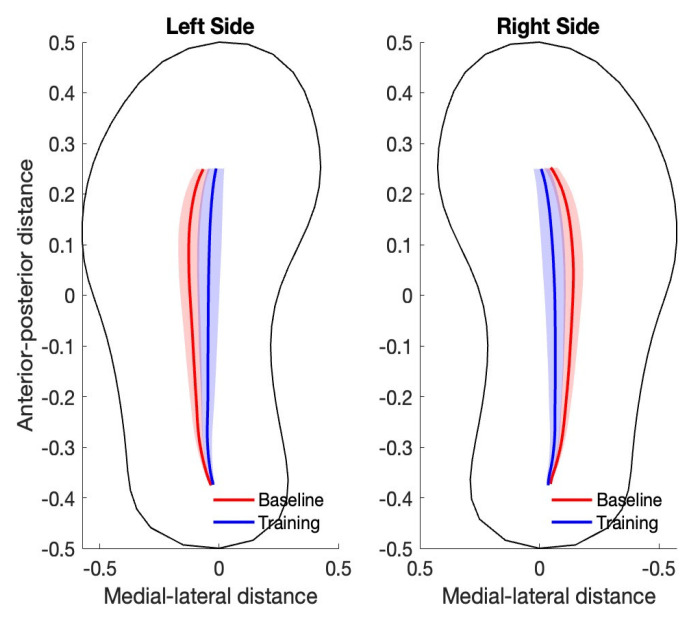
Gait line at the baseline (measured at warmup) and training with musical feedback. Shaded regions represent +/−1 SD.

There was a significant medial shift in the gait line when the participants practiced with the musical feedback. After SPM analysis was applied to the gait line, a significant difference was found between the baseline (measured at warm-up) and training with the musical feedback (see Figure 4). The mean peak medial–lateral COP (COPy) significantly reduced, showing a more medial displacement after practicing with the musical feedback (Table 1). An analysis of the peak percentage of difference on the gait line revealed that subjects ranged from −2.3% to −19% (−9.38 ± 4.37%). These data show that all participants were able to comprehend the feedback and medialize their COP. The majority (15/20) of the participants had the peak percentage of difference occurring at the midfoot, and four had a mixture of peak occurring at the midfoot and forefoot, while the remaining one occurred at the forefoot.

**Figure 5:**
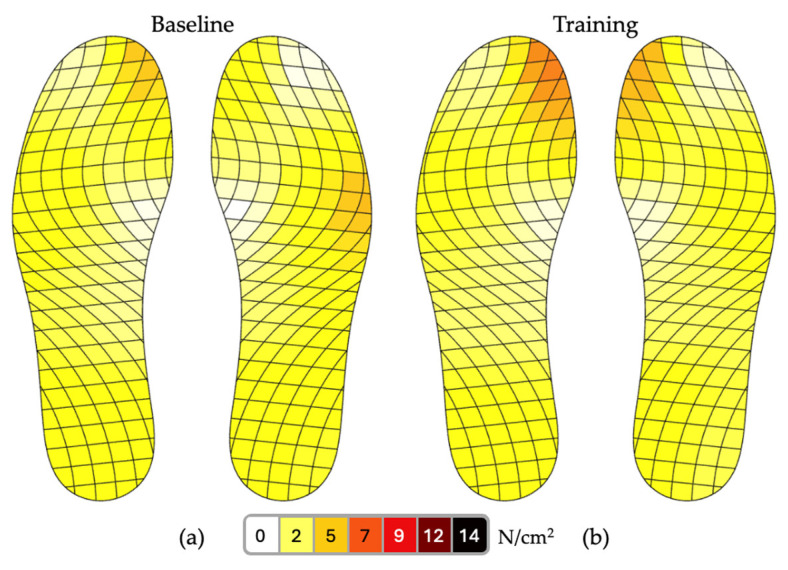
Mean plantar pressure throughout the stance phase at (**a**) the baseline and (**b**) training with musical feedback for one participant. Figure generated via a MOTICON OpenGo report.

There was a significant reduction (*p* < 0.01) in the mean plantar pressure during training with the musical feedback in the lateral aspect, as shown in Table 1. This plantar pressure decrease was observed on both sides. Conversely, a significant increase in the mean plantar pressure was observed in the medial aspect of both sides after training with the musical feedback. Figure 5 reveals a higher plantar pressure on the medial aspect of the foot throughout the stance phase when one participant practiced with the musical feedback.

### 3.2. Spatiotemporal Parameters

Data obtained through our motion capture system revealed that there was no significant change in any spatiotemporal parameters analyzed post-training when compared to the baseline after warm-up, as observed in Table 2.

### 3.3. Time to Accomplish Gait Modifications

The participants were not constrained by time when receiving instructions on the musical feedback and working to avoid muffling the sound. The majority of participants required less than five minutes to fully comprehend the musical feedback and successfully prevent the muffling of the song. On average, the participants spent 5:02 min exploring the feedback with a standard deviation of 4 min. The shortest duration recorded was 1:27 min, while the longest extended to 14:31 min.

### 3.4. Qualitative Rating

Fifteen participants completed the survey at the end of the testing session. All the participants agreed or strongly agreed that they “understood all the instructions that were explained during the test”, that they “could clearly detect changes in the sound”, that they “felt that the changes in the sound were related to the changes in how I walked”, and that they “enjoyed the music or sounds provided by the device”. For the questions regarding enjoying the device and the chances of using the device for rehabilitation, 13 out of 15 participants answered that they agreed or strongly agreed. Twelve participants said they would find it easy to implement the device for walking training in their weekly routine. Only three participants agreed or strongly agreed that the testing session was challenging, and one agreed that it was tiring. Seven out of the 15 participants agreed or strongly agreed that “walking with the musical feedback felt natural”. When we asked about discomfort or pain, eight participants referred to none, four mentioned some sort of soreness on the medial aspect of the foot, close to the arch, one reported slight friction of the foot on the back of the heel, another one said it was uncomfortable, and the remaining one pointed out some discomfort in the hips and feet. Aside from the questionnaire, one participant reported feeling, during the testing session, a slight discomfort with the mid-part of the insole on his left side, as if it was higher under his foot arch.

## 4. Discussion

Modifying gait patterns can be challenging without constant guidance from a physical therapist and a reliable measurement tool. In this study, we developed a musical feedback system that sonifies COP data to deliver personalized cues to the user in order to shift their gait line. Without specific verbal instructions on the feedback, all the participants were able to comprehend the musical feedback in a few minutes and avoid muffling the sound by medializing their plantar pressure. Therefore, our results indicate that our musical feedback system is capable of medializing plantar pressure and COP in healthy individuals.

Music was proven to elicit a variety of physiological responses, from relaxation to arousal [24,31], exerting a motivational influence on exercise, particularly in walking [32]. It has also been shown that auditory biofeedback can support a compromised proprioception system [33,34] and aid in movement planning and execution [13,33]. Therefore, sonification can have a beneficial impact on motor rehabilitation [25,35]. When compared with visual feedback, a common rehabilitation approach to biofeedback, auditory feedback does not restrict body movements [8], and it seems to be more effective for foot strike management in gait training [36,37]. Music has been explored in a few studies as a biofeedback approach. Neurological rehabilitation programs extensively use music either as a therapeutical approach or as a feedback tool [33,38]. However, a few published studies have approached orthopedic conditions through musical or auditory feedback [5,14,17,18,19,20,22]. Based on that, we designed a musical feedback system for future application in orthopedic clinical conditions, and in this study, we have shown evidence of a gait line shift through this system.

To our knowledge, this is the first study to modify plantar pressure with musical feedback. In previous studies, an audible beep was used to induce a medial shift in the plantar pressure of healthy participants [19,20], as well as for the rehabilitation of chronic ankle instability [22] and knee osteoarthritis [21]. An acoustic pattern that resembled the sound of walking on heavy snow for every instance of ground contact and a sequence of xylophone strikes for knee extension has been applied to achieve gait symmetry after hip arthroplasty. Lorenzoni et al., 2018 [39], combined music with pink noise to keep runners on a specific cadence. They found this method effective in increasing and decreasing cadence in runners. In a more recent study, Derie et al., 2021 [25,26] successfully employed music and noise to cue runners to reduce the momentary level of tibial shock. Other studies have used music to change either the gait [40,41] or a movement pattern [42] in healthy subjects. The musical feedback provided in this study—a sound filter applied to a playlist of music perceived as muffling—was designed to be agnostic to the music chosen by the user, such that patients could play any sound stream and still receive clear and understandable feedback about their walking. We consider this a strength of our system design. However, it is possible to use plantar pressure as music-generating input. In the aforementioned case study of a patient rehabbing from an ankle fracture [18], the sensors created music when sufficient pressure was generated and cycled through Pachelbel’s Canon in D. The canon only played when the task (reaching the same pressure during push-off as the contralateral, healthy foot) was completed successfully.

According to previous studies in healthy subjects [19,20] and preliminary data from a clinical trial with patients presenting with knee osteoarthritis [21], we chose a threshold of 25% of the peak COPy for the lowpass filter. In the aforementioned studies, the targeted threshold yielded a modification in the plantar pressure distribution that altered the knee loading for healthy participants but did so to a lesser extent in osteoarthritis patients. In the clinical trial, there were responders and non-responders, and responders demonstrated improvements in clinical outcomes. These findings support the investigation of gait line shifting to accomplish clinical outcomes, but caution should be taken with further assumptions. Further research is necessary with clinical populations that could benefit from gait line modification, such as patients with knee osteoarthritis, chronic ankle instability, and patellofemoral pain.

In the present experiment, we collected data from different time points during the whole testing session—warm-up, after warm-up, during training, and post-training. However, we only compared baseline data obtained during the warm-up with data from during training and data obtained after the warm-up with post-training data. The reasons for this were two-fold. The first reason was to ensure a reliable comparison by using the same equipment and similar testing conditions. In this case, both the warm-up and training lasted the same duration and utilized the same walking path (a figure-8 shape), which included accelerations and decelerations from the turns around the cones. Baseline data (after warm-up) with post-training data obtained during the walking trials were also comparable—a series of five trials on a straight line during which data were collected for one complete gait cycle, avoiding any acceleration or deceleration. The second reason was to understand the changes in gait parameters without the influence of music, solely from the musical feedback practice. From previous studies, it is known that there is a tendency for listeners to match their cadence (steps per minute) with the music tempo (beats per minute) [32,43]. Therefore, for spatiotemporal parameters, only data from the optical system was presented, even though the insoles were validated for that purpose [44].

The musical feedback system used in this study can be classified as discreet and easy to wear. All studies conducted in our laboratory with auditory feedback [18,19,20] implemented a wireless pressure-sensing insole that was slid inside common athletic shoes and remained unnoticeable to a casual viewer. Except for one participant who reported feeling the insole too high under his left foot arch, all the participants in this study had no problems with the insoles and the standardized shoes. In the Donovan et al. 2016 [22] study, the sensor was also inserted inside the patient’s shoes; however, the shoe had to be cut to place the sensor. For runners, the sensor was placed on the shoe, either on the insole or the cushion, but it was attached to the audio biofeedback device [45] or connected to a microcontroller on the arm [36]. For patients with hip and knee arthroplasty, a few inertial measurement units (IMUs) were applied for data capture to provide feedback [5,14,17], which could be troublesome for clinical applications. In other designs, only one IMU was attached to the tibia for running [25,46], and one was attached at the sternum to improve balance [47], which makes it more suitable for clinical purposes, even though it is still visible. One review highlighted that most sonification systems integrate a motion capture system with a smart device that will provide acoustic feedback, which prevents its usage in a clinical setting [11]. Our system comprises a shoe insole, a smartphone, and a desktop computer. Current work is in progress to eliminate the need for a desktop computer by integrating the musical feedback into a smartphone application.

One interesting aspect of the present study was the role of music familiarization in facilitating the shift in the center of pressure (COP). We recognize that, for the participant to perceive the muffling of the sound, they must have prior knowledge of the specific song being played. To support this, we provided participants with a diverse list of popular songs across a variety of genres. The participants were allowed to choose the songs they wished to listen to throughout the testing session. However, one participant, who was visiting from outside the United States, did not recognize any of the songs provided and, consequently, could not perceive the muffling of the sound when it occurred. In this instance, a song in his native language with which he was familiar was played for the instructions and comprehension of the new gait pattern. Following this, he successfully altered his gait line and reported his ability to recognize the change in the song.

Regarding the user experience, our study assessed comprehension, perceptibility, enjoyment, physical demand, discomfort, and the potential to adopt the system as a treatment tool. Previous studies that collected user experience data were performed in neurological rehabilitation settings [48,49,50]. Donovan et.al, 2016 [22] did not formally measure comfort; they mentioned that patients reported not feeling the sensor and that they “felt comfortable with walking in the manner to stop the device from eliciting the noise”.

While this study presents an innovative approach to gait line shifting for gait training, the findings should be interpreted with caution due to the small sample size and the inclusion of only healthy participants. We aimed to target COP gait alterations with musical feedback; however, we employed it for healthy younger adults who appeared to have a normal gait line. Future research is required to determine whether a specific clinical population, such as chronic ankle instability, will elicit the same results. Some further limitations of the present study must be acknowledged. Above, it was mentioned that there was no comparison across the four time points when data were collected. This configures a limitation to the fact that plantar pressure and COP data were not collected at the same time as the spatiotemporal parameters. Another limitation of this study is the short period of training, which could have limited the time for the participants to learn a new gait pattern. Since our goal was to determine whether the musical feedback was sufficient for the participants to modify their gait line, longer training was not required. In addition, in our previous study, the patient practiced for 12 min, over six sessions, and improvements were seen even 10 months after the treatment [18].

Because the system makes use of wireless insole sensors connected to a smartphone, the mobile musical feedback gait retraining system has the potential to assuage some barriers related to access to physical therapy healthcare. Thus, further work should focus on the system’s portability, as well as its feasibility for home-based training. Our study focused on observing the musical feedback’s immediate effect on the gait line; therefore, future research should explore the long-term effects in a follow-up. Additionally, further studies should be performed to apply this musical feedback system to a clinical population, such as patients with chronic ankle instability, patellofemoral pain, or other conditions that present with an altered center of pressure (COP).

## 5. Conclusions

The results of this study suggest that the real-time musical feedback system provided sufficient and clear cues to allow participants to shift their gait line medially. In this respect, this study indicates the potential for real-time auditory feedback in physical therapy and orthopedics, particularly if coupled with cloud-enabled telemetric health data monitoring.

## Figures and Tables

**Table 1:** Mean peak center of pressure (COP) and plantar pressure distribution along medial and lateral compartments during baseline (during warm-up) and training conditions.

	Baseline (Warm-Up)				Training			
	Left		Right		Left		Right	
Mean Peak COPy [−Lat/+Med]	−0.14 ± 0.04		−0.15 ± 0.03		−0.07 ± 0.03 **		−0.08 ± 0.03 **	
COPx at Mean Peak COPy [−Post/+Ant]	0.03 ± 0.12		−0.01 ± 0.12		−0.08 ± 0.24		−0.10 ± 0.15 **	
	Lateral	Medial	Lateral	Medial	Lateral	Medial	Lateral	Medial
Mean Plantar Pressure (N/cm^2^)	15.93 ±2.46	15.83 ± 2.09	16.15 ± 2.46	15.34 ± 2.85	12.50 ± 3.19 **	19.13 ± 2.66 **	12.70 ± 2.09 **	18.69 ± 2.96 **

COP: center of pressure; * *p* < 0.05; ** *p* < 0.01.

**Table 2:** Spatiotemporal gait parameters post-warm-up and post-training. CI: confidence interval.

	Post-Warm-Up	Post-Training	Mean Diff. (95% CI)	*p*-Value
Speed (m/s)	1.35 ± 0.18	1.36 ± 0.19	0.01 (−0.11, 0.13)	0.67
Cadence (Strides/min)	55.31 ± 3.46	55.32 ± 3.57	0.01 (−2.32, 2.34)	0.98
Stride Length (m)	1.47 ± 0.15	1.48 ± 0.13	0.01 (−0.08, 0.10)	0.91
Swing Time (s)	0.41 ± 0.02	0.41 ± 0.02	0 (−0.01, 0.01)	0.31
Stance Time (s)	0.68 ± 0.05	0.68 ± 0.05	0 (−0.03, 0.03)	0.53

## Data Availability

Data are available upon reasonable request.

## Supplementary Materials

The following supporting information can be downloaded at https://www.mdpi.com/article/10.3390/healthcare13020144/s1: Material S1: Playlist of popular songs available for the participants to choose from. Material S2: Qualitative questionnaire applied to participants after the testing session. Video S1: Representation of the musical feedback with shifts in plantar pressure while standing using a song generated via Udio AI Music Generator (udio.com; accessed on 25 November 2024) for demonstration purposes. Video S2: Representation of the musical feedback with shifts in plantar pressure while standing using a song generated via Udio AI Music Generator (udio.com, accessed on 25 November 2024) for demonstration purposes.
