# Supplementary material for: Exploring Musical Feedback for Gait Retraining: A Novel Approach to Orthopedic Rehabilitation
Source: Healthcare (Basel). 2025 Jan 14;13(2):144. doi: 10.3390/healthcare13020144 (PMC11764814; doi:10.3390/healthcare13020144)
Supplement: Supplementary file 1 [file healthcare-13-00144-s001.zip › healthcare-3377879-supplementary.pdf]

Supplemental materials were placed under link:

https://zenodo.org/records/14262370
